# The Methanol Extract of *Angelica sinensis* Induces Cell Apoptosis and Suppresses Tumor Growth in Human Malignant Brain Tumors

**DOI:** 10.1155/2013/394636

**Published:** 2013-11-10

**Authors:** Yu-Ling Lin, Wen-Lin Lai, Horng-jyh Harn, Pei-Hsiu Hung, Ming-Chang Hsieh, Kai-Fu Chang, Xiao-Fan Huang, Kuang-Wen Liao, Ming-Shih Lee, Nu-Man Tsai

**Affiliations:** ^1^College of Biological Science and Technology, National Chiao Tung University, Hsinchu 30010, Taiwan; ^2^Center for Bioinformatics Research, National Chiao Tung University, Hsinchu 30010, Taiwan; ^3^School of Medical Laboratory and Biotechnology, Chung Shan Medical University, Taichung 40201, Taiwan; ^4^Clinical Laboratory, Chung Shan Medical University Hospital, Taichung 40201, Taiwan; ^5^Department of Pathology, China Medical University Hospital, Taichung 40402, Taiwan; ^6^Department of Traditional Chinese Medicin, Ditmanson Medical Foundation Chia-Yi Christian Hospital, Chiayi 60002, Taiwan; ^7^Department of Bioindustry Technology, Da-Yeh University, Changhua 51591, Taiwan; ^8^Institute of Molecular Medicine and Bioengineering, National Chiao Tung University, Hsinchu 30010, Taiwan

## Abstract

Glioblastoma multiforme (GBM) is a highly vascularized and invasive neoplasm. The methanol extract of *Angelica sinensis* (AS-M) is commonly used in traditional Chinese medicine to treat several diseases, such as gastric mucosal damage, hepatic injury, menopausal symptoms, and chronic glomerulonephritis. AS-M also displays potency in suppressing the growth of malignant brain tumor cells. The growth suppression of malignant brain tumor cells by AS-M results from cell cycle arrest and apoptosis. AS-M upregulates expression of cyclin kinase inhibitors, including p16, to decrease the phosphorylation of Rb proteins, resulting in arrest at the G_0_-G_1_ phase. The expression of the p53 protein is increased by AS-M and correlates with activation of apoptosis-associated proteins. Therefore, the apoptosis of cancer cells induced by AS-M may be triggered through the p53 pathway. In *in vivo* studies, AS-M not only suppresses the growth of human malignant brain tumors but also significantly prolongs patient survival. In addition, AS-M has potent anticancer effects involving cell cycle arrest, apoptosis, and antiangiogenesis. The *in vitro* and *in vivo* anticancer effects of AS-M indicate that this extract warrants further investigation and potential development as a new antibrain tumor agent, providing new hope for the chemotherapy of malignant brain cancer.

## 1. Introduction

Glioblastoma multiforme (GBM), the most common malignant tumor of the central nervous system, is a highly vascularized and invasive neoplasm. The annual incidence of GBM was approximately 5–7 per 100,000 people per year in the USA between 1995 and 2008 [1]. Because of its malignant properties, rapid growth, diffuse invasion, and resistance to current therapies, the median survival of GBM patients is approximately 50 weeks [2]. Current treatments combine surgery, radiation, and chemoradiotherapy, providing an increase in the median overall survival from 12 to 15 months [3, 4]. Ongoing preclinical and clinical studies evaluating the efficacy of antitumor drugs provide hope for increasing survival time [3].

 In the clinic, various chemotherapeutic drugs have been used for GBM therapy, such as carmustine (BCNU) [5, 6], temozolomide (TMZ) [5–7], and bevacizumab (Avastin) [8, 9]. However, the use of a single agent for newly diagnosed or recurrent GBM does not effectively improve treatment efficacy [3, 10]. In previous clinical studies, monotherapy using temsirolimus (CCI-779), a small-molecule inhibitor of the mammalian target of rapamycin (mTOR), was found to promote 6-month progression-free survival (PFS6) rates of 2.3–7.8% in phase II trials [11, 12]. The standard therapy of adding the adjuvant sorafenib to the treatment of newly diagnosed GBM patients also does not improve treatment efficacy [13]. Combining chemotherapeutic drugs that inhibit parallel pathways has been considered to improve the effectiveness of targeted molecular therapy. However, no patients receiving a combination therapy of temsirolimus and sorafenib were shown to remain progression free at 6 months [14]. Thus, a drug with multi-targeted inhibition in different pathways is one strategy to improve the effectiveness of chemotherapy.

*Angelicae sinensis radix *(AS) is commonly used in traditional Chinese medicine to treat several diseases, such as menopausal symptoms [15, 16], gastric mucosal damage [17–20] and hepatic injury [21–23]. Previous literatures have shown that AS improves the level of therapeutic angiogenesis for the treatment of ischemic disease [24, 25]. Ferulic acid is a critical component of AS that induces angiogenesis by promoting endothelial cell proliferation through upregulating cyclin D1 and VEGF [26]. Additionally, various components, such as Z-ligustilide, neodiligustilide, alkylphthalide and n-butylidenephthalide (BP), have cytotoxic activities in tumor cells [27, 28]. BP suppresses tumor cell proliferation, upregulates the expression of cyclin kinase inhibitors, including p21 and p27, to decrease the phosphorylation of Rb proteins, and downregulates cell cycle regulators, resulting in cell arrest at G_0_-G_1_ phase [28, 29]. BP also shows a dramatic antitumor effect on growth resting and apoptosis in GBM tumors *in vitro* and *in vivo*, and both p53-dependent and -independent pathways of apoptosis were found to be involved in its cytotoxicity mechanism [28]. BP represses the transcriptional activity of hTERT by downregulating Sp1 expression [30]. BP also exerts antiangiogenic activity by inhibition of cell cycle progression and induction of apoptosis in endothelial cells [31]. However, unknown components with multiple antitumor functions may exist in different AS extracts.

In this study, we examined the cytotoxic effect of AS-M on GBM cells. AS-M induced cell cycle arrest at the G_0_-G_1_ phase by upregulating the expression of p16. AS-M induced apoptosis through the p53 pathway, which was associated with increased expression of the p53 protein. In *in vivo* studies, AS-M not only suppresses the growth of human malignant brain tumors but also significantly prolongs patient survival. In addition, AS-M has potent anticancer effects involving cell cycle arrest, apoptosis, and antiangiogenesis.

## 2. Materials and Methods

### 2.1. Preparation of Methanol Extracts

**Figure 1:**
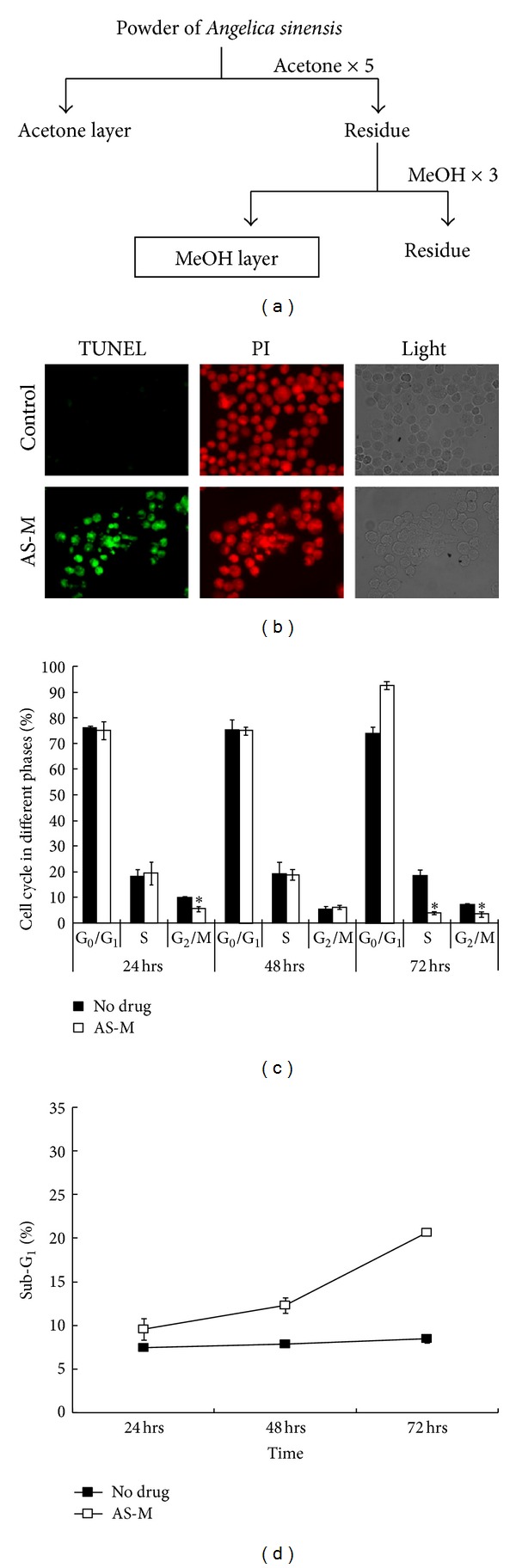
The cytotoxic effects of AS-M on GBM cells. (a) The methanol extraction procedure for the roots of *Angelica sinensis.* (b) AS-M induced cell apoptosis. After 72 h of treatment, apoptotic cells were detected by fluorescence microscopy (×400) using in situ TUNEL staining and propidium iodide counterstaining. (c) AS-M enhanced cell cycle arrest at the G_0_-G_1_ phase. (d) AS-M increased the sub-G_1_ cells. DBTRG-05MG cells were treated with AS-M or control medium for 0, 24, 48, and 72 hours, washed, and stained with propidium iodide. Each column represents the mean ± SD; *P < 0.05.

The roots of *radix Angelicae sinensis *were supplied by Chung-Yuan Co. (Taipei, Taiwan) and were identified by Professor Han-Ching Lin. A voucher herbarium specimen was deposited at the School of Pharmacy at the National Defense Medical Center (Taipei, Taiwan). Dried and powdered rhizomes of *radix Angelicae sinensis *(2.0 kg) were extracted with acetone (5 L, 3 times) and methanol (5 L, 3 times), respectively. The flow chart of the extraction protocol is shown in Figure 1. The extracts were concentrated under reduced pressure to yield an acetone extract (AS-A) and a methanol extract (AS-M). These extracts were dissolved in DMSO and incubated with shaking at 25°C for 1 hour. The extracts were stored at 4°C before each *in vitro* experiment.

### 2.2. Cell Lines and Cell Culture

The DBTRG-05MG human GBM cell line and BALB/3T3 mouse fibroblast cells were obtained from the American Type Culture Collection (Rockville, MD). G5T/VGH human GBM cells and N18 mouse neuroblastoma cells were obtained from the Bioresources Collection and Research Center (Hsinchu, Taiwan). The DBTRG-05MG cells were maintained in RPMI 1640 containing 10% fetal bovine serum at 37°C in a humidified atmosphere containing 5% CO_2_. The G5T/VGH, N18, and BALB/3T3 cells were cultured in DMEM containing 10% fetal bovine serum at 37°C in a humidified atmosphere containing 5% CO_2_. 

### 2.3. Analysis of Cell Cytotoxicity

Cell viability was evaluated using a modified 3-(4,5-dimethylthiazol-2-yl)-2,5-diphenyltetrazolium bromide (MTT) assay. Briefly, cells were incubated in 96-well plates (5 × 10^3^ cells/well) containing 100 **μ**L growth medium. The cells were grown for 24 h before treatment with 100 **μ**L herbal extracts dissolved in medium (0–500 **μ**g/mL). The final concentration of DMSO in each preparation was approximately 0.01%. After 72 h of incubation, the drug-containing medium was replaced with 50 **μ**L fresh medium containing MTT (400 **μ**g/mL; Sigma) for 6 to 8 h. The MTT medium was then removed, and 100 **μ**L DMSO was added to each well. The absorbance of the dissolved solutions was detected at 550 nm using an MRX Microtiter Plate Luminometer (DYNEX, Sunnyvale, CA). The absorbance value of untreated control cells was considered to be 100%. The IC50 was defined as the concentration resulting in a 50% absorbance decrease in the drug-treated cells compared with the untreated cells.

### 2.4. Cell Cycle Analysis

DBTRG-05MG cells were treated with the AS-M extract (70 **μ**g/mL) for 24, 48, or 72 h. Cell cycle analysis was evaluated by DNA staining with propidium iodide (PI) using flow cytometry. Briefly, 2 × 10^6^ adherent cells were detached by trypsinization and then resuspended in 0.8 mL PBS. A total of 200 **μ**L PI solution (50 **μ**g/mL PI + 0.05 mg/mL RNase A; Sigma) was added, and the cells were incubated at 4°C overnight. A total of 2 × 10^4^ cells were analyzed for FL2 intensity using a FACScan flow cytometer (Becton Dickinson Immunocytometry Systems, San Jose, CA) and CellQuest analysis software (Becton Dickinson Immunocytometry Systems). G_0_-G_1_ phase was gated in M_1_ (%G_0_-G_1_ phase = M_1_ × 2), G_2_-M phase was gated in M_2_ (%G_2_-M phase = M_2_ × 2), total cells were gated in M_3_ (%S phase = M_3_ − [(M_1_ × 2) + (M_2_ × 2)]), and sub-G_1_ phase was gated in M_4_.

### 2.5. Terminal Deoxynucleotidyl Transferase-Mediated Nick End Labeling Assay (TUNEL Assay)

Apoptotic cell death was assayed for the drug-treated cells using an In Situ Cell Death Detection Kit, POD (Roche, Mannheim, Germany), according to the manufacturer's instructions. Briefly, cells were cultured on culture dishes and analyzed at the indicated time points (0, 6, 12, 24, 48, and 72 h) after AS-M (70 **μ**g/mL) treatment. For the AS-M-treated cell group, the suspended cells were collected. For the control group, the adherent and floating cells were collected. The cells were fixed with 3.7% formaldehyde at room temperature for 15 min on silane-coated glass slides (Muto Pure Chemicals, Tokyo, Japan), washed once with PBS, and incubated in cold permeabilization solution (0.1% Triton X-100 + 0.1% sodium citrate) after reducing the activity of endogenous peroxidase with 3% H_2_O_2_. The cells were washed with PBS again and incubated with the TUNEL reaction mixture for 60 min at 37°C. After another wash with PBS, counterstaining with PI was performed for the cell count determination. To quantify the level of apoptosis, the resulting slides were viewed under a fluorescence microscope (Nikon, Kawasaki, Japan). For the histologic TUNEL staining of GBM tumor tissues (s.c. or i.c. GBM tumors with or without AS-M treatment), the tumors were harvested and fixed with 10% neutral formalin. Paraffin-embedded sections (7 **μ**m per section) of the tumors were stained using an In Situ Cell Death Detection Kit, POD. After dewaxing, rehydration, and proteinase digestion, the slides were incubated with 100 **μ**L TUNEL reaction mixture and covered with a lid for 1 h at 37°C in a humidified atmosphere in the dark. Finally, the slides were washed three times with PBS, mounted, and visualized under a fluorescence microscope.

### 2.6. Immunocytochemistry Staining

Cells were placed on coverslips, cultured in a six-well, plate and then subjected to the appropriate treatment. After treatment, the cells were fixed with 4% paraformaldehyde, and their membranes were ruptured with Triton X-100. They were blocked with 5% skim milk for 1 h at 37°C and then incubated with the respective antibodies, including anti-p16, anti-p53, anti-Bax, anti-Bcl2, anticytochrome *c*, anti-AIF, anticaspase-9, and anticaspase-3 (1/100 dilution; Santa Cruz Biotechnology, Inc., Santa Cruz, CA), overnight at 4°C, followed by incubation with the appropriate horseradish peroxidase-conjugated anti-mouse or anti-rabbit IgG secondary antibody (1/1000 dilution; Santa Cruz Biotechnology) for 1 h at 37°C. Finally, the coverslips were observed and photographed under a light microscope at a magnification of ×400.

### 2.7. Animal Studies

The DBTRG-05MG human GBM cells were used in animal experiments to monitor the antitumor activity of AS-M. Male Foxn1 nu/nu mice (10–12 weeks old) were obtained from the National Laboratory Animal Center (Taipei, Taiwan). All procedures were in compliance with the standard operating procedures of the Laboratory Animal Center of Tzu Chi University (Hualien, Taiwan). Nude mice (six mice per group) were implanted s.c. with 5 × 10^6^ DBTRG-05MG cells. The tumor-bearing animals were treated with AS-M (s.c. injection of 500 mg AS-M/kg/d; AS-M group) or vehicle (s.c. injection; control group) 3, 6, and 9 days after tumor cell implantation. Tumor size was measured with a caliper, and tumor volume was calculated as L × H × W × 0.5236. The animals were sacrificed when the tumor volume exceeded 1,000 mm^3^. Tumor and normal tissue sections were observed and photographed under a light microscope at a magnification of ×400.

### 2.8. H&E Staining

For the histological H&E staining of GBM tumor and normal tissues with or without AS-M treatment, the tissues were harvested and fixed with 10% neutral formalin. After dehydrating the tissues and embedding them in paraffin wax, tissue sections (4 **μ**m/section) were placed on clean glass slides and dehydrated in an oven for 30 min at 60°C. Prior to staining, the tissue slides were deparaffinized, rehydrated, and then stained with Mayer's hematoxylin and eosin Y solution for 3 minutes. After air-drying, the tissue slides were mounted with mounting media and visualized under a Nikon light microscope camera system.

### 2.9. Immunohistochemical Staining

Paraffin-embedded sections were obtained from the tumors (s.c. GBM tumors with or without AS-M treatment) and were processed for immunohistochemical staining. Briefly, the slides were treated with 3% hydrogen peroxide in 1 × PBS for 10 min to block endogenous peroxidase activity after being dewaxed and rehydrated. Next, the sections were washed three times with TBS-T (1 × TBS containing 0.05% Tween 20) for 5 min each time, and nonspecific antibody binding was blocked by 10% fetal bovine serum in PBS for 10 min at room temperature. The sections were incubated with a goat polyclonal anti-Ki-67 antibody (1/100 dilution; Santa Cruz Biotechnology) at 4°C overnight, and the immune complexes were visualized using a horseradish peroxidase-conjugated anti-goat IgG secondary antibody (1/1,000 dilution; Santa Cruz Biotechnology). Alternatively, the sections were incubated with a rabbit polyclonal anticleaved caspase-3 (Asp175) antibody (1/1,000 dilution; Cell Signaling Technology) at 4°C overnight. The LSAB2 system (DAKO, Carpinteria, CA) was used to visualize the immune complexes by incubating the sections with 0.5 mg/mL diaminobenzidine and 0.03% (v/v) H_2_O_2_ in PBS for 10 min. Finally, the sections were counterstained with hematoxylin, mounted, observed under a light microscope at a magnification of ×400, and photographed. 

### 2.10. Statistics

Data are expressed as means ± SD. Statistical significance was analyzed using Student's *t* test. Survival analysis was performed using the Kaplan-Meier method. *P* < 0.05 was considered to be statistically significant.

## 3. Results

### 3.1. Cytotoxic Effects of AS-M on Tumor Cells and Normal Cells

The antiproliferative effects of AS-M on GBM cells, neuroblastoma cells, and normal fibroblasts were determined. The IC_50_ values of AS-M after a 48 h incubation with the brain tumor cells (IC_50_ = 21–40 **μ**g/mL) were significantly lower than the values for the normal cells (IC_50_ > 400 **μ**g/mL, *P* < 0.001; Table 1). Therefore, these results show that AS-M induces high cytotoxicity in brain tumor cells but no cytotoxicity in normal cells. After AS-M treatment, the GBM tumor cells were analyzed by TUNEL staining, and the results showed that the AS-M-treated, detached GBM cells were undergoing apoptosis at 72 h (Figure 1(b)).

### 3.2. Effects of AS-M on the Cell Cycle in GBM Cells

Cell cycle analysis of DBTRG-05MG cells showed that treatment with 70 **μ**g/mL AS-M resulted in cell cycle arrest at G_0_-G_1_ phase (>90%; Figure 1(c)) and sub-G_1_ phase (Figure 1(d)). At 72 h, AS-M induced a significant proportion of cells to arrest at G_0_-G_1_ phase, which was accompanied by a concurrent decrease in the proportion of cells in S and G_2_-M phase (*P* < 0.05). In addition, AS-M also decreased the proportion of DBTRG-05MG cells that entered G_2_-M phase at 24 h after treatment. 

### 3.3. Apoptotic Pathways Induced by AS-M in GBM Cells

**Figure 2:**
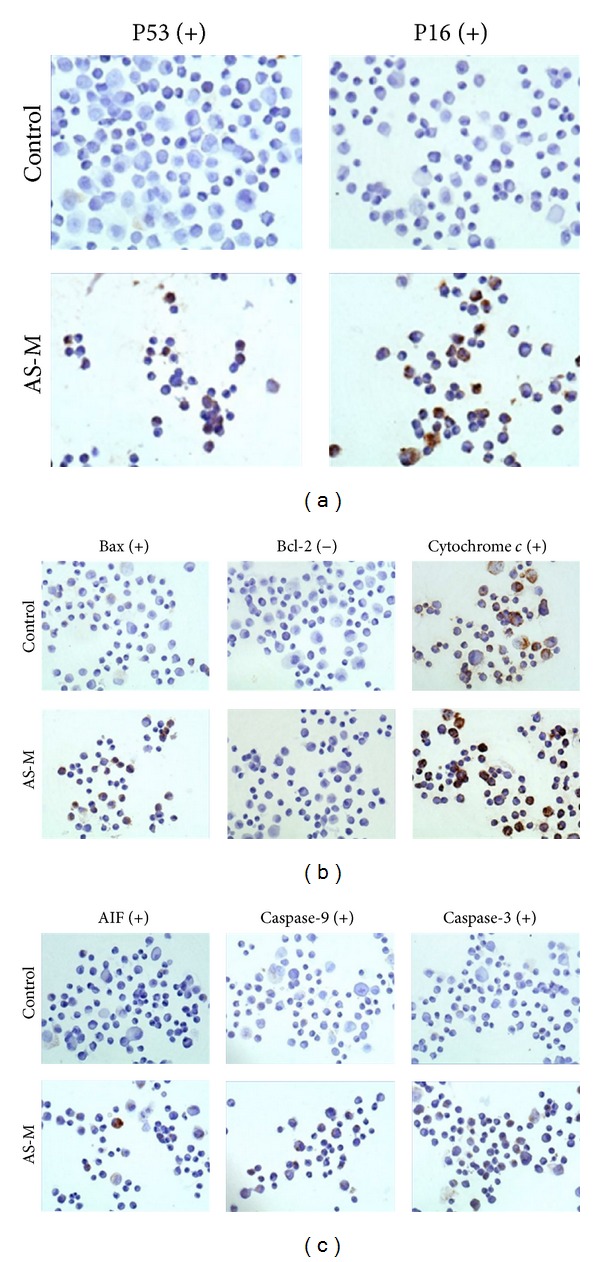
AS-M induced apoptosis through the activation of multiple pathways. DBTRG-05MG cells were treated with AS-M (50 **μ**g/mL) for 48 hours. Apoptosis markers were detected and analyzed by ICC using specific antibodies against (a) p16 and p53 for detection of the cell cycle pathway and (b)-(c) Bax, Bcl-2, cytochrome c, AIF, caspase-9, and caspase-3 for detection of the mitochondria-related apoptosis pathway.

To investigate the apoptotic pathways induced by AS-M treatment, the expression of the p53 and p16 proteins was first evaluated by immunocytochemistry. AS-M increased the expression of p53 and p16 (Figure 2(a)), indicating that AS-M can trigger the cell cycle checkpoint machinery. The protein expression of Bax, cytochrome *c*, AIF, and Bcl-2 was also measured in AS-M-treated DBTRG-05MG cells. The expression of Bax, cytochrome *c*, and AIF increased and the expression of Bcl-2 decreased in these cells (Figure 2(b)). In addition, the activities of caspase-9 and caspase 3 were determined (Figure 2(c)). Both caspase 9 and caspase 3 were activated in the DBTRG-05MG cells after AS-M treatment.

### 3.4. Antitumor Effects of AS-M on Xenograft Tumor Growth

**Figure 3:**
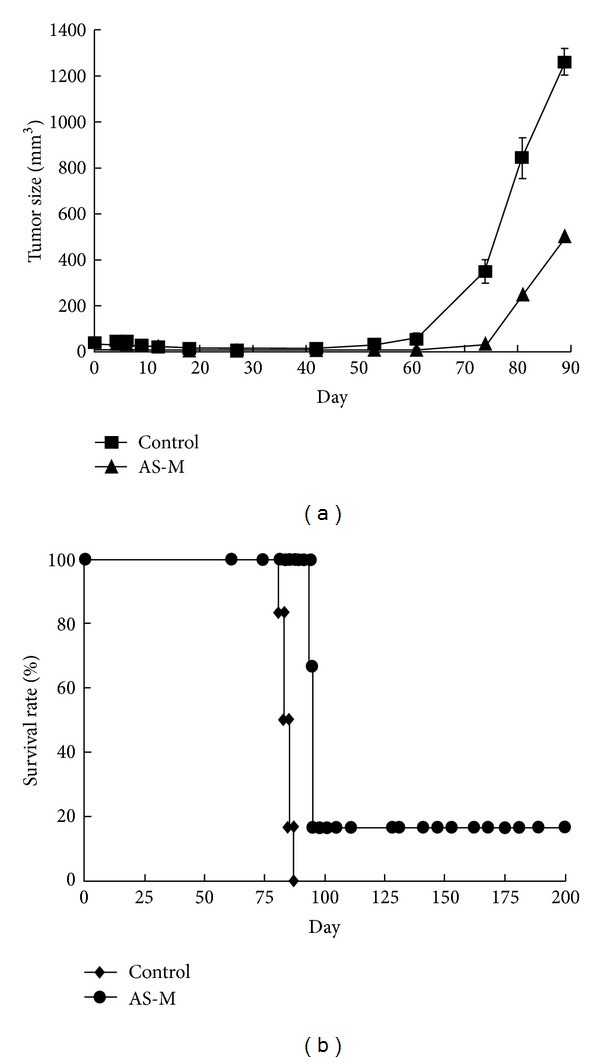
AS-M suppressed tumor growth and prolonged the animal survival rate in mice subcutaneously implanted with human GBM cells. DBTRG-05MG cells were implanted s.c. into the hind flank region of Foxn1 nu/nu mice. The mice were treated with vehicle as a control or AS-M (500 mg/kg/day) at days 3, 6, and 9. (a) AS-M inhibited the tumor growth and (b) prolonged the survival rate in these mice. The mice were sacrificed when the tumor size exceeded 1000 mm^3^. The tumor size values are means ± SE.

To determine whether BP can suppress human GBM tumor growth, nude mice were inoculated with human DBTRG-05MG cells and treated with 500 mg/kg AS-M on days 4, 5, 6, 7, and 8. Significant suppression of tumor growth with respect to the untreated group was observed in the AS-M-treated mice (Figure 3(a)). The mean tumor size at day 89 was >1000 mm^3^ in the control group and 503 mm^3^ in the AS-M-treated group (Figure 3(a); *P* < 0.05). Survival was significantly prolonged in the AS-M-treated nude mice compared to the control group (Figure 3(b); *P* < 0.001). At day 200, the survival rates were 0% (0/6) and 16.7% (1/6) for the untreated and AS-M-treated groups, respectively. 

### 3.5. Antitumor Activity of AS-M on Human GBM *In Vivo*

**Figure 4:**
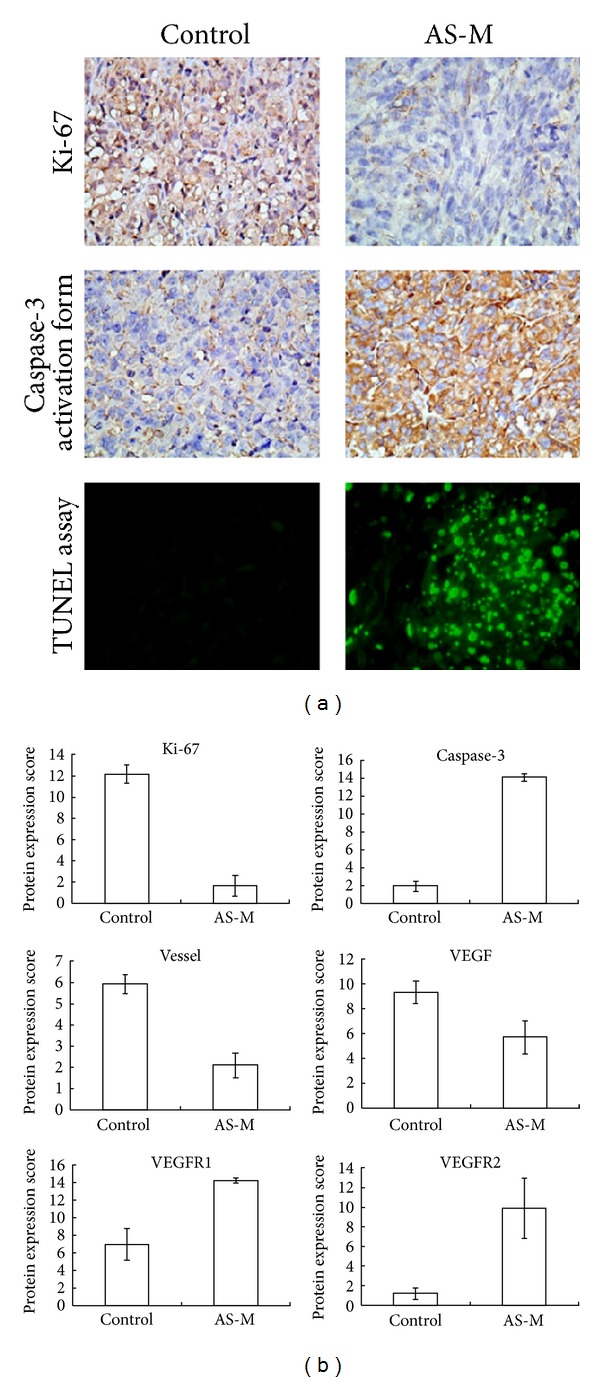
AS-M suppressed the growth of the human GBM tumors by inducing cell apoptosis, cell cycle arrest, and antiangiogenesis *in vivo*. (a) Immunohistochemical staining and the TUNEL assay were performed on the GBM tumor tissues. Representative photographs of sections from the control group and AS-M-treated group are shown. In the immunohistochemistry experiment, GBM tumors were stained for the cell proliferation marker Ki-67 and the cell apoptosis marker cleaved caspase-3. In the TUNEL assay, the tumors were stained to show the DNA fragmentation of apoptotic cells. The Ki-67- and caspase-3-positive cells are shown in brown, and the TUNEL-positive cells are shown in green (×400). Quantification of protein expression was performed using Quick score, which multiplies the staining intensity by the percentage of positive cells. (b) The mean values for the Ki-67, caspase-3, VEGF, VEGFR1, and VEGFR2 levels and the vessel number were calculated for the tumors treated with AS-M. All the data in AS-M group is significant difference from the control group (*P* < 0.05).

To confirm whether AS-M can induce human GBM tumor cell death *in vivo*, the cytotoxic activity of AS-M on GBM tumors in a nude mouse model was examined. Histologic analysis showed that the human GBM tissues treated with AS-M *in vivo* displayed decreased Ki-67 expression, increased caspase-3 activity, and tumor cell apoptosis (Figure 4(a)). AS-M also decreased the vessel number and expression of VEGF but enhanced the expression of VEGFR1 and VEGFR2 (Figure 4(b)).

### 3.6. Effects of AS-M on Tissue Damage

**Figure 5:**
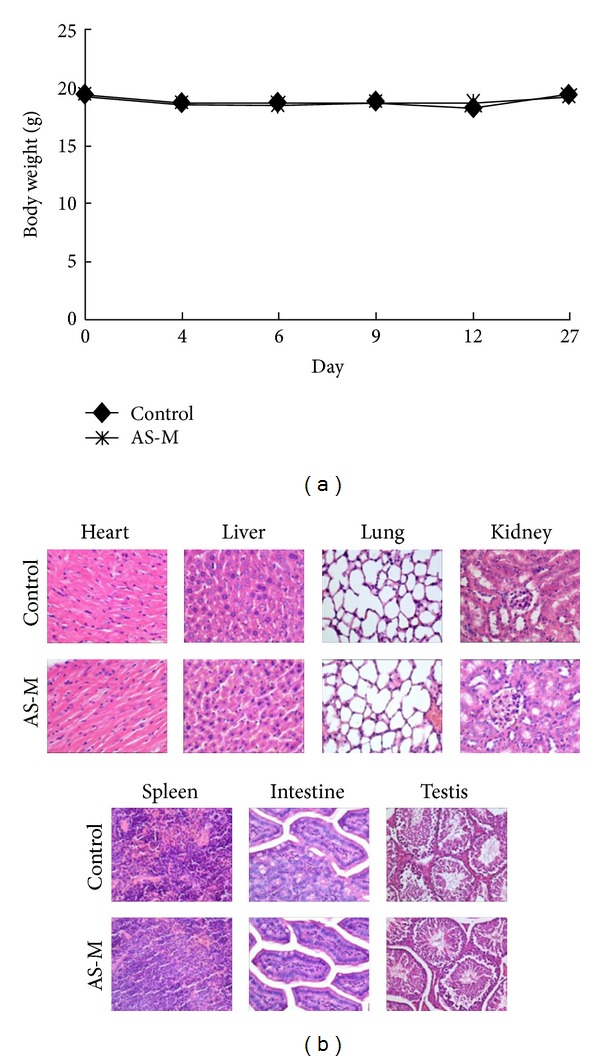
AS-M showed no or low cytotoxicity in normal tissue. (a) The body weights of the mice in the control and AS-M groups were not significantly different. The body weights are represented as means SEM. (b) Long-term treatment of AS-M was safe in mice bearing tumor. In histological analyses, the heart, liver, spleen, intestine, lung, kidney, and testis were evaluated by H&E staining (×400).

We next examined the effects of AS-M on normal organs to investigate the safety of AS-M treatment. As the results show, mice treated with AS-M did not show changes in body weight (Figure 5(a)) or organ morphology, including that of the liver, heart, spleen, lung, and kidney, compared to untreated mice (Figure 5(b)). Furthermore, AS-M treatment did not result in changes in the levels of WBCs, platelets, lymphocytes, BUN, creatinine, GOT, and GPT in the sera, revealing that AS-M treatment does not affect hepatic, renal, and immune system functions (Figure  S1; see Supplementary material available on line at http://dx.doi.org/10.1155/2013/394636).

## 4. Discussion

*A. sinensis* has been used for a long time in traditional Chinese medicine for its biological activities, such as immune system regulation [32, 33], menopausal symptom relief [15, 16], and cardioprotection [34, 35]. The different extracts of *A. sinensis*, such as water, chloroform, and acetone extracts, have demonstrated antitumor biofunctions [36, 37]. In this study, AS-M has demonstrated to be a potential antitumor extract isolated from* A. sinensis *that efficiently inhibits GBM tumor growth. In an* in vitro* cytotoxic assay, brain tumor cells were sensitive to AS-M and normal fibroblast cells were unsusceptible to AS-M (Table 1). AS-M dramatically inhibited 90% of the subcutaneous tumor growth (Figure 3(a)) and prolonged survival *in vivo*. AS-M efficiently suppressed tumor growth by inducing cell cycle arrest at the G_0_-G_1_ phase and promoting apoptosis (Figure 1). The AS-M mechanism was found to involve the cyclin/CDK/CKI cell cycle regulatory system and the upregulation of p16 and p53 expression. The tumor suppressor protein p53 is essential for the G_1_ DNA damage checkpoint [38]. An increase in the p53 protein level causes a downregulation of cell division [38]. The p16 protein exerts a tumor suppressor function by binding to the cyclin D_1_ CDK4/CDK6 complex, preventing the phosphorylation of the retinoblastoma (Rb) protein and resulting in G_1_ arrest [39, 40]. AS-M triggered the expression of CDK inhibitors, thus decreasing the activity of the cyclin/CDK complex and preventing Rb phosphorylation.

The p53-dependent apoptosis pathway may be involved in the mechanism of AS-M-induced apoptosis in GBM tumor cells. The p53 protein directly activates the proapoptotic Bcl-2 family member Bax, which results in cytochrome *c* release, loss of mitochondrial membrane potential, and caspase-9 and caspase-3 processing. The phosphorylation of p53 by AS-M may abolish the Mdm2 inhibition, leading to an increase in Bax transcription (Figure 2(b)) and inhibition of Bcl-2 transcription. When the Bax/Bcl-2 level is imbalanced, Bax can form a homodimer to trigger mitochondrial permeabilization, cytochrome *c* release (Figure 2(b)), and procaspase-9 and procaspase-3 cleavage (Figure 2(c)), subsequently resulting in apoptosis. Additionally, AS-M was found to upregulate AIF expression (Figure 2(c)). AIF has a key role in the caspase-independent apoptosis pathway [41]. When the mitochondrial outer membrane is permeabilized, AIF translocates to the cytosol and nucleus, where it induces chromatin condensation and DNA fragmentation [41]. Therefore, AS-M triggers both p53-dependent and caspase-independent pathways for apoptosis.

Local administration, such as intratumoral or intracranial injection, is one of the therapeutic methods of brain tumor in clinical [42–44]. This therapy is an alternative to vascular administration that bypasses the BBB and delivers therapeutic agents directly into the brain [45]. In this study, we designed the athymic mice were which implanted s.c. with GBM tumor and were treated s.c. with AS-M to mimic the therapy of intratumoral injection. These results provided that amounts of AS-M accumulated in tumor area and led to the maximum antitumor effects of cytotoxic chemotherapy. 

*A. sinensis* has been demonstrated to exert biological functions on the vascular system. The water extract of *A. sinensis* has angiogenesis-promoting effects, as it increases VEGF expression and stimulates JNK 1/2 and p38 phosphorylation to enhance endothelial cell proliferation. In contrast to previous literature, AS-M inhibited tumor growth* in vivo* via its antiproliferative and antiangiogenic activities on tumor cells in this study. AS-M suppressed angiogenic vessel growth by inhibiting the expression of VEGF, which plays a critical role in inducing the formation of tumor vasculature (Figure 4(b)). Yeh et al. indicated that n-butylidenephthalide exhibited antiangiogenic activity by activating the p38 and ERK 1/2 signaling pathways [31]. These findings suggest that there are antiangiogenic compounds similar to BP in AS-M. AS-M treatment upregulated the expression of VEGFR1 and VEGFR2 (Figure 4(b)). Tumors treated with chemotherapy have been showed that these antitumor drugs can reduce the levels of VEGF expressed by tumor cells [31]. In such condition, VEGFs contributed as mitogenic factors to tumor cells in addition to their angiogenic functions [46, 47]. Therefore, tumor may overexpress VEGFR to more efficiently get the VEGF as the reduction of VEGF levels.

## 5. Conclusion

In this study, AS-M has promising anticancer properties due to its ability to induce cell cycle arrest and to promote apoptosis in GBM cells. AS-M likely contributes to the anticancer and antiangiogenesis properties described here and may provide new hope for the effective chemotherapy of malignant brain tumors.

## Supplementary Material

While the results refer to the anti-tumor activity of AS-M in GBM tumor, the safety of AS-M treatment must be evaluated. The blood biochemical indexes of animals treated with AS-M were analyzed to evaluate the organ damages after AS-M treatment.

## Figures and Tables

**Table 1:** The IC50 of AS-M in brain tumors and normal cells.

Cell line	Tumor type	IC_50_ of AS-M (*μ*g/mL)
Brain tumors		
DBTRG-05MG	Human GBM cell	25.5 ± 11.0*
G5T/VGH	Human GBM cell	21.1 ± 6.8*
N18	Mouse neuroblastoma	40.3 ± 1.3*
Normal cells		
Balb/3T3	Mouse fibroblast cell	>400

Note: values are mean ± SD IC_50_ (*μ*g/mL) after treatment for 48 h.

*Significant difference from the brain tumors versus normal cells of AS-M treatment (*P* < 0.001).
