# Supplementary material for: The Methanol Extract of Angelica sinensis Induces Cell Apoptosis and Suppresses Tumor Growth in Human Malignant Brain Tumors
Source: Evid Based Complement Alternat Med. 2013 Nov 10;2013:394636. doi: 10.1155/2013/394636 (PMC3844186; doi:10.1155/2013/394636)
Supplement: Supplementary file 1 — While the results refer to the anti-tumor activity of AS-M in GBM tumor, the safety of AS-M treatment must be evaluated. The blood biochemical indexes of animals treated with AS-M were analyzed to evaluate the organ damages after AS-M treatment. [file 394636.f1.pdf]

Supplementary materials

Blood biochemical index

Wistar rats (males, 350-370 g BW, n=6) were treated with 1 g/kg AS-M, blood samples were collected at 0, 0.5, 1, 3, 6, 9, 12, 18, 24, 36, 48, and 72 h. Blood samples were collected and immediately centrifuged at 3,000 × g for 10 min. Blood urea nitrogen (BUN), creatinine, glutamate oxaloacetate transaminase (GOT), glutamate pyruvate transaminase (GPT), white blood cells (WBC), platelets, and lymphocytes were measured with an autoanalyzer (Vitros 750, Johnson & Johnson Co., NY, USA) to evaluate various organ functions. BUN and creatinine are measurements of renal function. GOP and GPT are commonly measured in clinical settings as a part of a diagnostic liver function test. WBC and lymphocyte counts are measurements of immune system function.

A

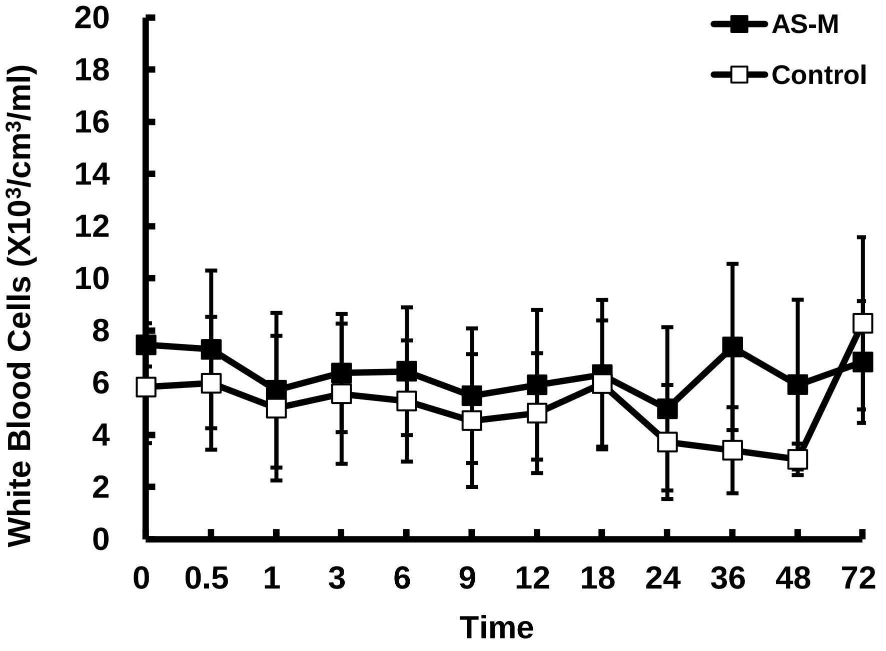

AS-M
Control
White Blood Cells (X10³/cm³/ml)
20
18
16
14
12
10
8
6
4
2
0
0
0.5
1
3
6
9
12
18
24
36
48
72
Time

B

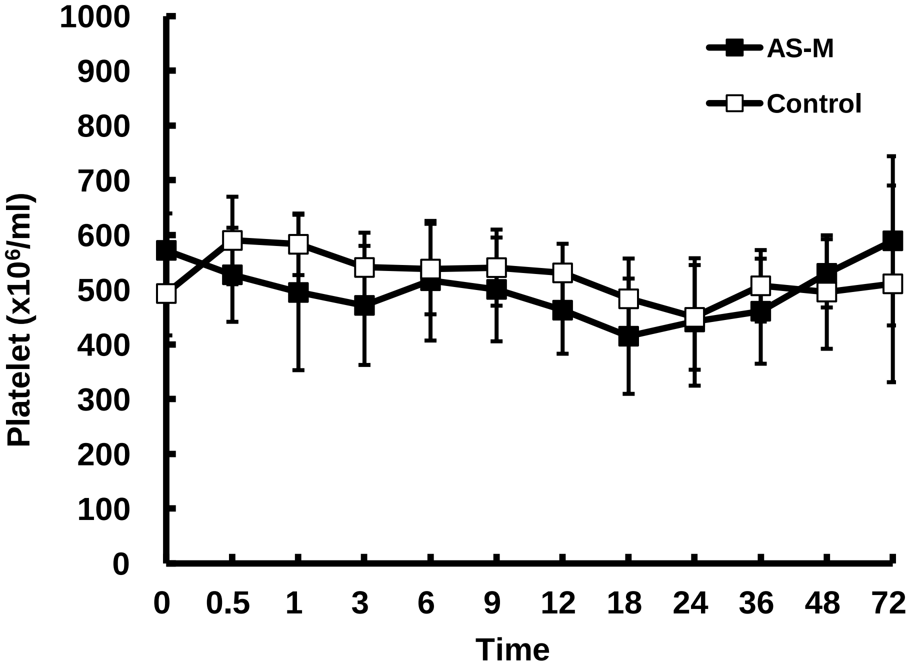

AS-M
Control
Platelet (x10⁶/ml)
1000
900
800
700
600
500
400
300
200
100
0
0
0.5
1
3
6
9
12
18
24
36
48
72
Time

C

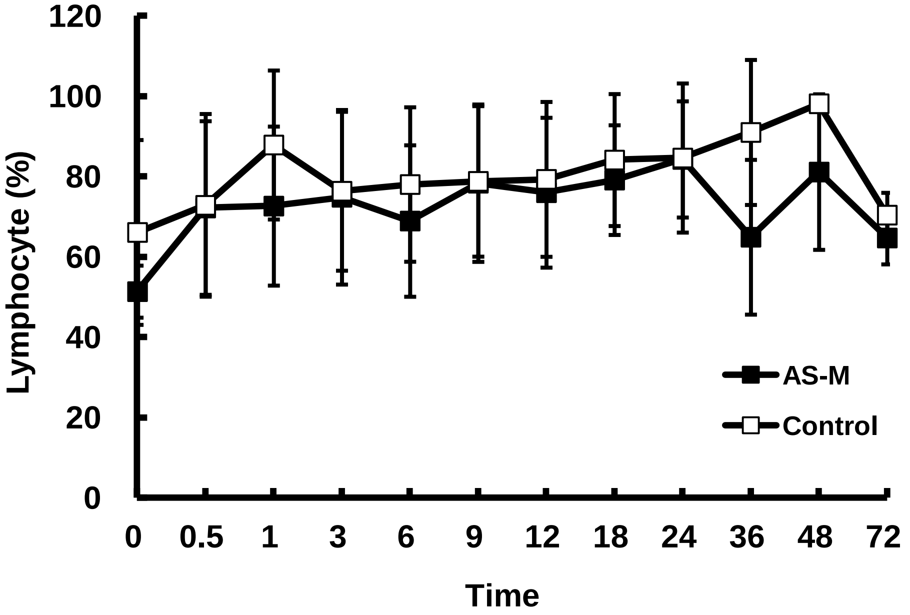
Lymphocyte (%)
120
100
80
60
40
20
0
0
0.5
1
3
6
9
12
18
24
36
48
72
Time
AS-M
Control

D

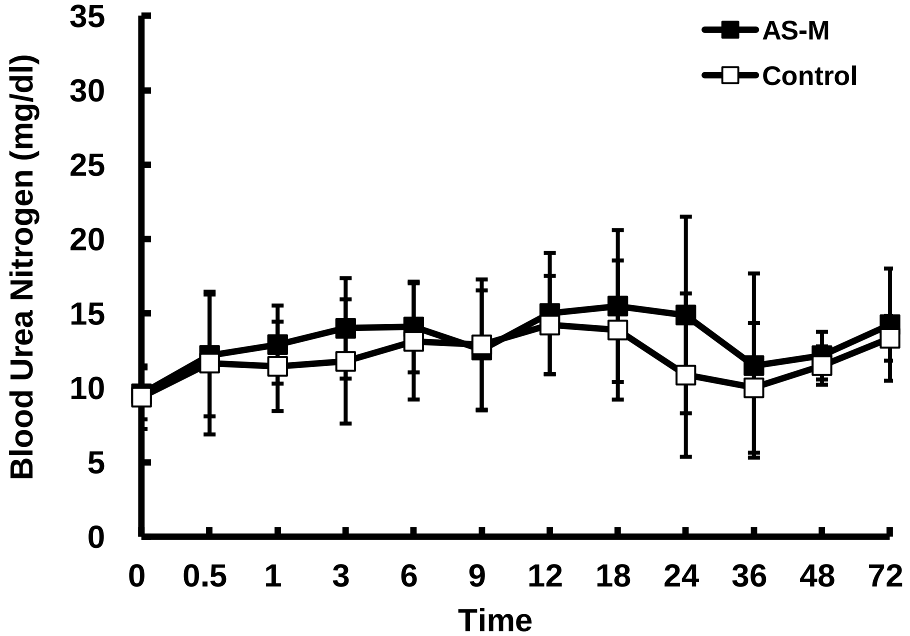
Blood Urea Nitrogen (mg/dl)
35
30
25
20
15
10
5
0
0
0.5
1
3
6
9
12
18
24
36
48
72
Time
AS-M
Control

E

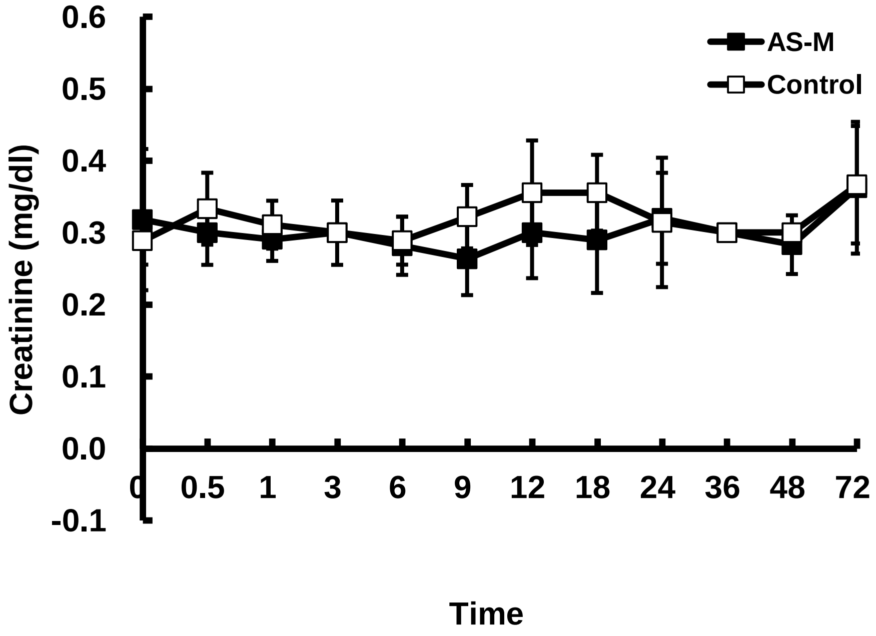

0.6
0.5
0.4
0.3
0.2
0.1
0.0
-0.1
Creatinine (mg/dl)
AS-M
Control
0
0.5
1
3
6
9
12
18
24
36
48
72
Time

F

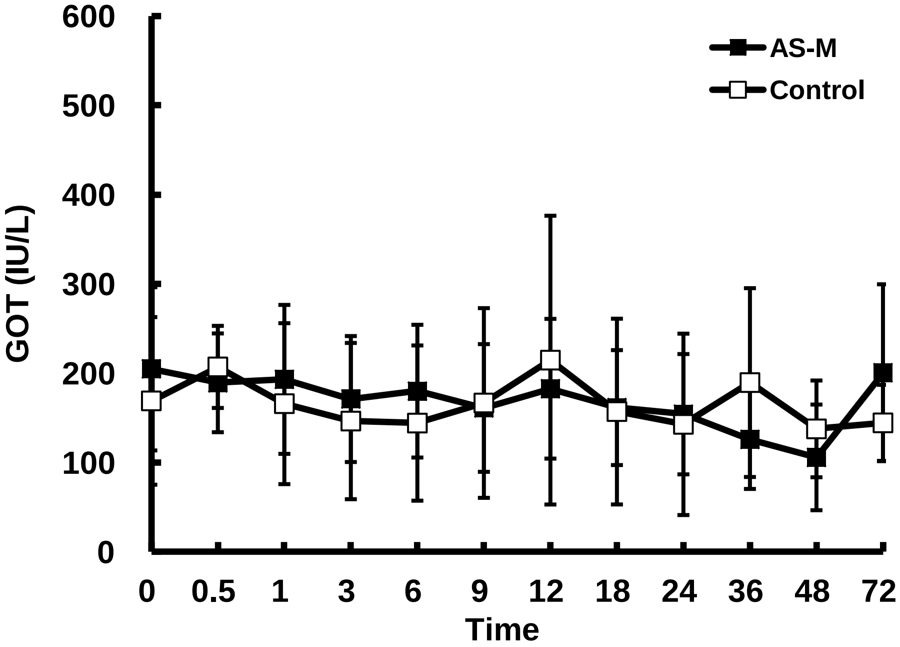

600
500
400
300
200
100
0
GOT (IU/L)
AS-M
Control
0
0.5
1
3
6
9
12
18
24
36
48
72
Time

G

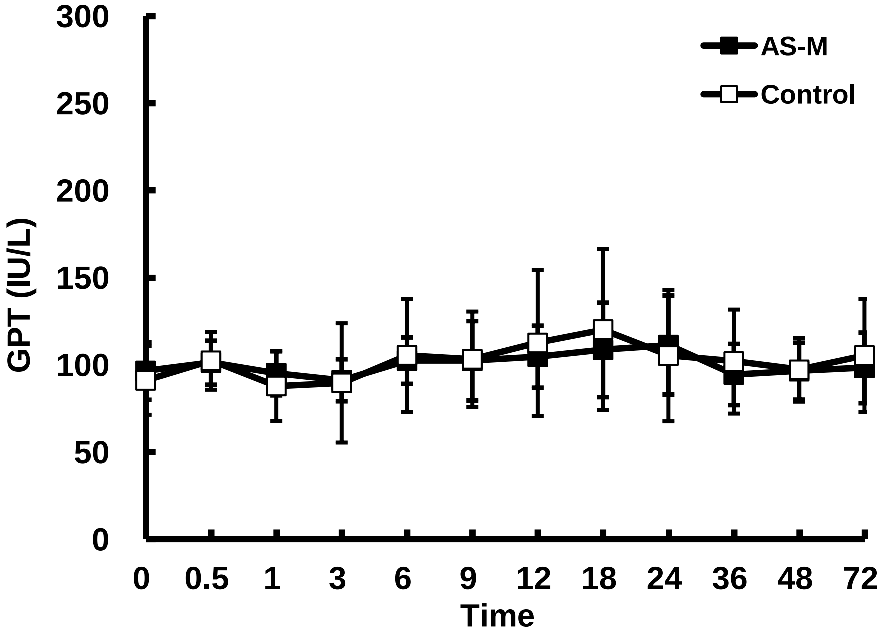

Figure S1. The serum levels of (A) WBC, (B) platelet, (C) lymphocyte, (D) BUN, (E) creatinine, (F) GOT, and (G) GPT in AS-M-treated rats. All values are mean ± SD of three independent experiment (n=10)
